# Health-related quality of life of cranial WHO grade I meningioma patients: are current questionnaires relevant?

**DOI:** 10.1007/s00701-017-3332-8

**Published:** 2017-09-27

**Authors:** Amir H. Zamanipoor Najafabadi, Marthe C. M. Peeters, Daniel J. Lobatto, Marieke L. D. Broekman, Timothy R. Smith, Nienke R. Biermasz, Saskia M. Peerdeman, Wilco C. Peul, Martin J. B. Taphoorn, Wouter R. van Furth, Linda Dirven

**Affiliations:** 1 Department of Neurosurgery, Leiden University Medical Center, Postal Zone J11-R, Albinusdreef 2, 2333ZA Leiden, The Netherlands; 2 Department of Neurology, Leiden University Medical Center, Leiden, The Netherlands; 3 Department of Neurosurgery, University Medical Center Utrecht, Utrecht, The Netherlands; 4 Department of Neurosurgery, Cushing Neurosurgery Outcomes Center, Brigham and Women’s Hospital, Harvard Medical School, Boston, MA USA; 5 Department of Endocrinology, Leiden University Medical Center, Leiden, The Netherlands; 6 Department of Neurosurgery, VU Medical Center, Amsterdam, The Netherlands; 7 Department of Neurosurgery, Haaglanden Medical Center, The Hague, The Netherlands; 8 Department of Neurology, Haaglanden Medical Center, The Hague, The Netherlands

**Keywords:** Meningioma, Health-related quality of life, Questionnaires, Content validity

## Abstract

**Background:**

The clinical relevance of Health-Related Quality of Life (HRQoL) in meningioma patients has been increasingly acknowledged in recent years. Various questionnaires have been used. However, almost none of these questionnaires has been particularly developed for and/or validated in this patient group. Therefore, the aim of this study was to assess the relevance and comprehensiveness of existing HRQoL questionnaires used in meningioma research and to assess the agreement between patients and health care professionals (HCPs) on the most relevant and important HRQoL issues.

**Methods:**

A systematic literature search, following the PRISMA statement, was conducted to identify all HRQoL questionnaires used in meningioma research. Semi-structured interviews were organized with patients and HCPs to (1) assess the relevance of all issues covered by the questionnaires (score 0–3: not relevant–highly relevant), (2) assess the ten most important issues, and (3) identify new relevant HRQoL issues.

**Results:**

Fourteen different questionnaires were found in the literature, comprising 140 unique issues. Interviews were conducted with 20 patients (median age 57, 71% female) and 10 HCPs (4 neurosurgeons, 2 neurologists, 2 radiotherapists, 1 rehabilitation specialist, 1 neuropsychologist; median experience 13 years). Meningioma patients rated 17–80% of the issues in each of the questionnaires as relevant, HCPs 90–100%. Patients and HCPs agreed on the relevance of only 49 issues (35%, Cohen’s kappa: 0.027). Both patients and HCPs considered lack of energy the most important issue. Patients and HCPs suggested five additional relevant issues not covered by current HRQoL questionnaires.

**Conclusions:**

Existing HRQoL questionnaires currently used in meningioma patients do not fully cover all relevant issues to these patients. Agreement between patients and HCPs on the relevance of issues was poor. Both findings support the need to develop and validate a meningioma-specific HRQoL questionnaire.

**Electronic supplementary material:**

The online version of this article (10.1007/s00701-017-3332-8) contains supplementary material, which is available to authorized users.

## Introduction

Meningioma is the most prevalent (53.4%) type of benign central nervous system tumor with an incidence of 7.86 per 100.000 person years [28]. As these tumors originate from the arachnoid cap cells, the majority of tumors are supratentorial (90%) [33]. In general, patients have a near normal life expectancy after surgery and/or radiotherapy [28, 39]. However, based on the location of the tumor, patients may suffer from a wide variety of signs and symptoms and problems in the physical, psychological and social domains, even in the long term after intervention [33, 44].

Patient function can be categorized into three distinct levels, as described by the World Health Organization International Classification of Functioning, Disability and Health (ICF, 2001) criteria: impairment (e.g., visual problems), activity limitations (e.g., not able to drive because of physical problems) and participation restrictions (e.g., not able to work). A Health-Related Quality of Life (HRQoL) instrument is a multidimensional outcome measure, including domains on physical, psychological and social functioning as well as symptoms induced by the disease and its treatment, thereby covering function on all three ICF levels [14].

HRQoL data can be physician-, proxy- or patient-reported, but the use of patient-reported outcome measures (PROM), reflecting the patient’s perspective, has been increasing in the last decade [10, 44]. Indeed, patients are thought to be the best source to rate their own health status [14]. HRQoL can be measured using more generic (e.g., SF-36, EQ-5D, MDASI), cancer specific (e.g., FACT-G, EORTC QLQ-C30) [1, 42] or disease-specific questionnaires (e.g., FACT-BR, EORTC QLQ-BN20, MDASI-BT) [3, 36, 42] and can be used in both clinical research and daily practice. In clinical research, HRQoL questionnaires can be used as primary or secondary outcome measures, which in combination with survival rates can be used to measure the net clinical benefit of different treatment modalities [12]. Treatment either improves or worsens the duration and quality of (progression-free) survival, but the effect on both is not necessarily the same. When duration and quality of life are affected in opposite directions, a trade-off discussion arises [11]. In clinical practice, HRQoL questionnaires can be used as a facilitating tool for patient-doctor communication, for monitoring patients’ problems and functioning during the disease trajectory and as a quality indicator of healthcare [21].

While the number of meningioma studies using HRQoL questionnaires as the primary or secondary outcome measure has increased in the last decade, it is remarkable to note that almost all existing HRQoL questionnaires used in meningioma research were not developed and/or validated in earlier series with this condition [1, 22, 36, 41]. It may therefore be questioned whether the issues addressed in these questionnaires are relevant for meningioma patients and whether these questionnaires cover the entire spectrum of issues and symptoms of this patient group. Validation of these questionnaires in meningioma patients is therefore needed to assess whether all items are applicable to meningioma patients, but also to assess the performance (i.e., measurement properties) of the PROM in the target population. At the moment, multiple questionnaires may be needed to comprehensively cover all issues relevant for meningioma patients.

The aim of this study was to assess whether existing HRQoL questionnaires used in cranial meningioma research indeed cover issues that are relevant for meningioma patients and whether relevant problems/issues are missing (i.e., content validity). In addition, we aimed to assess the agreement between patients and physicians with respect to the most relevant and important issues for meningioma patients.

## Methods and materials

A literature search was conducted to identify all HRQoL questionnaires used in clinical research with meningioma patients. Issues covered by these questionnaires were categorized into different HRQoL domains, which were subsequently used in semi-structured interviews with both patients and health care professionals (HCPs). The aim of these semi-structured interviews was to assess the content validity (i.e., the degree to which the content of existing questionnaires is an adequate reflection of the HRQoL of meningioma patients) and consisted of three parts: (1) to identify all relevant HRQoL issues (the interviews continued until no new issues arose), (2) to determine the relevance of all issues identified in the literature search, including those in the existing HRQoL questionnaires, and (3) to determine the ten most important HRQoL issues.

### Literature study

A literature search was conducted in the following electronical databases: Embase, MEDLINE, Web of Science, CINAHL, PsychInfo, Academic Search Premier, COCHRANE and ScienceDirect up to October 2015, according to the Preferred Reporting Items for Systematic Reviews and Meta-Analyses (PRISMA) statement [24]. Search terms used were “meningioma,” “quality of life” and terms formulated to exclude case reports and studies with animals only (see supplementary Table 1 for the formal search strategy). Reference lists of included articles were scanned for additional studies. Inclusion criteria were the following: original peer-reviewed articles including HRQoL questionnaires as outcome measures in adult meningioma patients. Exclusion criteria were as follows: articles not in English and studies with animals. Two independent reviewers (AHZN and MCMP) screened all titles and abstracts for HRQoL questionnaires. HRQoL domains and issues covered by these questionnaires were categorized by one researcher (AHZN) and verified independently by two other researchers (LD, WRvF). Disagreement was resolved with discussion and consensus.

### Semi-structured interviews with patients and healthcare professionals

#### Subject selection

A convenient number of patients, randomly selected, were eligible for inclusion if clinically diagnosed (symptoms and imaging) with a benign intracranial meningioma (WHO grade I) for which they visited the neurosurgery outpatient clinic in the Leiden University Medical Center (LUMC) between 2011 and 2015. Patients were older than 18 years and fluent in Dutch. Both patients with a convexity meningioma and with a skull base meningioma, irrespective of previous anti-tumor therapy (surgery and/or radiotherapy), were included to reflect the heterogeneity of this patient group. Similarly, patients before treatment, short term after treatment (up to 2 years after surgery) and long term after treatment (at least 2 years after surgery) were included. Patients were interviewed only once. Patients were excluded when histopathological diagnosis revealed that the tumor was not a benign meningioma (all patients had been surgically treated prior to analysis), diagnosed with neurofibromatosis type 2 or they had a history of tumors of the central nervous system other than benign meningioma. HCPs were neurosurgeons, neurologists, radiotherapist, rehabilitation specialists and clinical psychologists who treated meningioma patients in their daily practice.

#### Semi-structured interviews

Semi-structured interviews were conducted by AHZN with both patients and HCPs, consisting of four steps: step 1: patients had to answer the open question “*What are your meningioma-related problems/issues at this moment?*” and HCPs had to answer the question “*Which problems/issues are relevant for meningioma patients?*”; step 2: HRQoL domains identified in the questionnaires were discussed to identify all relevant HRQoL issues for meningioma patients; step 3: patients and HCPs scored the relevance of each found issue on a 4-point Likert scale (0 = not relevant at all, 1 = of little relevance, 2 = quite relevant, 3 = highly relevant); step 4: patients and HCPs had to indicate which ten problems/issues they deemed most important. Relevance and importance were assessed by patients for themselves based on their experiences of the last month and by HCPs for meningioma patients in general.

### Data analysis

In step 1 and 2 of the interviews, issues and problems not covered by existing HRQoL questionnaires used in meningioma research were identified. In step 3, all HRQoL problems/issues covered by existing questionnaires were assessed for relevance: issues were considered relevant when ≥30% of the patients or ≥30% of the HCPs scored the issue as relevant (score 1–3) on the 4-point Likert scale. A cutoff of ≥ 30% was chosen because of the heterogeneity of the disease (e.g., based on tumor characteristics, patients are likely to assesses different issues as relevant) and variability due to the small number of participants. Agreement between patients and HCPs was assessed using Cohen’s kappa (degree of agreement: moderate 0.41–0.60, substantial 0.61–0.80, excellent 0.81–0.99) [40]. In addition, specific positive and negative agreements were assessed that described the probability of the described groups finding the same issue relevant or not relevant [8]. In step 4, HRQoL issues were considered important when at least 30% of patients or HCPs reported the issue as important. Results were compared between patients and HCPs, but also between patients with skull base and convexity meningiomas, and between patients before surgery, up to 2 years after surgery and patients followed for at least 2 years after surgery. Baseline characteristics and relevance and importance of HRQoL questionnaires and items were described using descriptive statistics. Descriptive statistics are presented as median and interquartile range (IQR) as data were skewed. To determine significant differences in baseline characteristics, Pearson’s chi-square and Fisher’s exact test were used for dichotomous outcomes and the Mann-Whitney U Test or Kruskal-Wallis test for continuous outcomes. All statistics were performed using IBM SPSS Statistics for Windows version 23.0 (Armonk, NY, USA), and *p*-values <0.05 were considered statistically significant.

### Ethics statement

This study was conducted after approval of our institutional review board. Informed consent was obtained before participation.

## Results

### Literature study

**Fig. 1:**
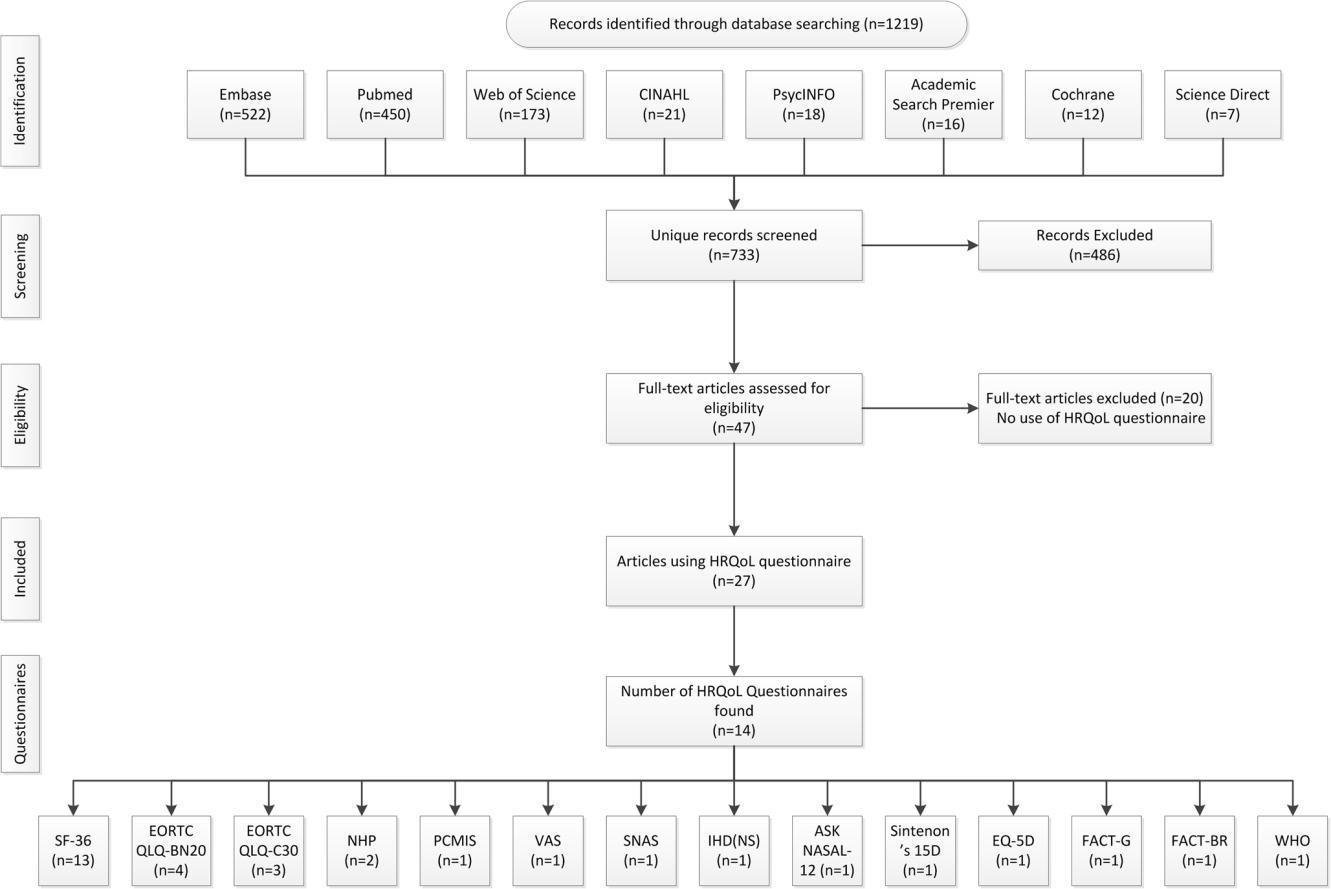
Flow chart of literature search and questionnaire selection

A total of 733 unique records were found, including 27 articles using HRQoL questionnaires in meningioma patients (Fig. 1). The following questionnaires were used: five generic HRQoL questionnaires (SF-36: *n* = 13; NHP: *n* = 2; Sintenon’s 15D: *n* = 1; EQ-5D: n = 1; WHOQOL: n = 1) [16, 31, 34, 38, 41], two disease-specific questionnaires for cancer patients (EORTC QLQ-C30: *n* = 3; FACT-G: *n* = 1) [1, 5], two disease-specific questionnaires for brain tumor patients (EORTC QLQ-BN20: n = 2; FACT-BR: *n* = 1) [27, 42], one disease-specific questionnaire for patients with advanced breast cancer (VAS: *n* = 1) [30], one disease-specific questionnaire for petroclival meningiomas (PCMIS: *n* = 1) [26], one disease-specific questionnaire for neurosurgically treated patients with central nervous system tumors (IHDNS: *n* = 1) [25], one disease-specific questionnaire for neuro-oncology tumors (SNAS: n = 1) [13] and one disease-specific questionnaire for patients with anterior skull base pathology (ASK Nasal-12: n = 1) [20]. Only the FACT-G and FACT-BR questionnaires have been validated in meningioma patients [5, 42]. Of the 439 items covered by the questionnaires, a total of 140 unique HRQoL issues were identified (i.e., many questionnaires covered the same issues or multiple items covered one issue in the same questionnaire).

### Subject characteristics

Subject characteristics are presented in Table 1. A total of 20 meningioma patients (75% females) were interviewed, with a median age of 57 years (IQR: 48-67): skull base (*n* = 10), convexity/cerebral falx (n = 10), before surgery (*n* = 5), up to 2 years after surgery (*n* = 9) and patients followed for at least 2 years after surgery (*n* = 6). Two patients received postsurgical radiotherapy. Baseline characteristics of subgroups are presented in supplementary tables 2 and 3. In addition, 10 HCPs (4 neurosurgeons, 2 neurologists, 1 rehabilitation specialist and 1 neuropsychologist; 30% female) were interviewed, with a median age of 50 years (IQR: 40–54). HCPs had a median experience of 13 years (IQR: 8–23), consulting a median of 25 (IQR: 10-40) new meningioma patients each year.

**Table 1:** Patient characteristics

Characteristics	All patients (*n* = 20)
Age in years at interview, median (IQR)	57 (48-67)
Sex, n (% female)	15 (75%)
Time since clinical diagnosis in months, median (IQR)	23 (5-51)
Karnofsky Performance Status, median (IQR)	95 (80–100)
Charlson Comorbidity Index, n (%)	
0	15 (75%)
1–2	4 (20%)
> 2	1 (5%)
Midline shift present, n (%)	4 (20%)
Edema present, n (%)	16 (80%)
Corticosteroid use, n (%)	3 (15%)
Antiepileptic drug use, n (%)	3 (15%)
Tumor location	
Convexity meningioma	10 (50%)
Skull base meningioma	10 (50%)
Moment of interview, n (%)	
Before surgery	5 (25%)
After surgery < 2 years	9 (45%)
After surgery ≥ 2 years	6 (30%)
Surgical resection, n (%)	15 (75%)
Simpson grade I–III	13 (87%)
Simpson grade IV–V	2 (13%)
Postsurgical radiotherapy, n (%)	2 (10%)

n: number. IQR: interquartile range

### Semi-structured interviews

#### Relevance of existing HRQoL questionnaires

**Fig. 2:**
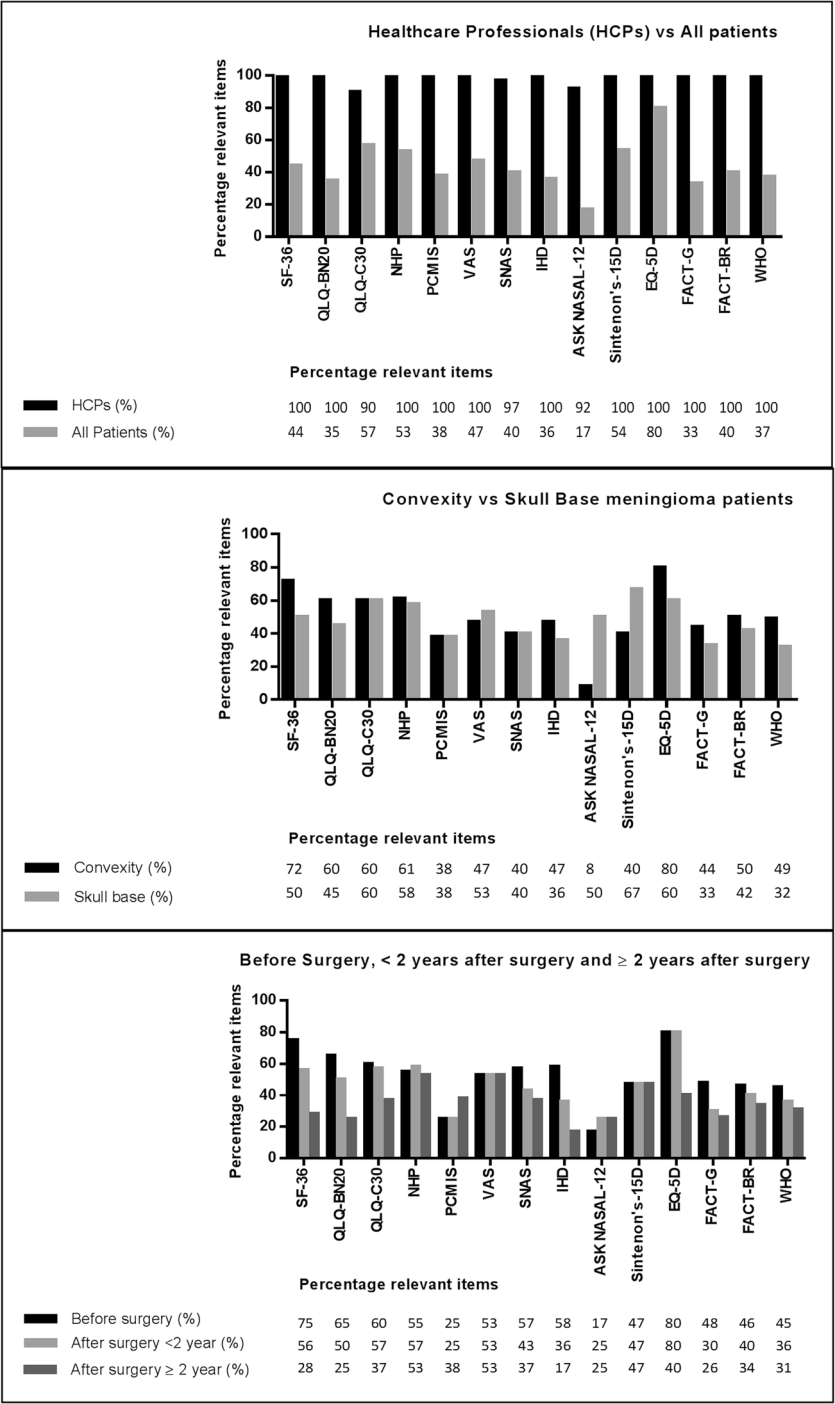
Relevance of health-related quality of life items in questionnaires used in meningioma research: percentages describe the proportion of items assessed as relevant per questionnaire

Meningioma patients assessed 45/140 (32%) issues as relevant, whereas HCPs assessed 136/140 (97%) issues as relevant. Meningioma patients and HCPs agreed on the relevance of 49 out of 140 issues (35%, Cohen’s kappa: 0.027). Specific positive agreement was 0.247, which means that the probability that patients and HCPs assess the same issues as relevant is 24.7%. The specific negative agreement, the probability that patients and HCPs assess the same issue as non-relevant, was 0.040 (4%), which is driven by the observation that physicians found almost all items relevant. When analyzing the results per questionnaire, meningioma patients rated 17-80% of the issues in the questionnaires as relevant with the ASK NASAL-12 as least relevant (17%) and the EQ-5D as most relevant (80%). HCPs on the other hand rated 90–100% of the issues as relevant with the EORTC QLQ-C30 as least relevant (90%) and the EORTC QLQ-BN20, SF-36, PCMIS, VAS, IHD(NS), NHP, Sintenon’s 15D, WHOQOL, EQ-5D, FACT-G and FACT-BR as most relevant (all 100%). Convexity meningioma patients rated 8–80% of the issues in the questionnaires as relevant (least relevant: ASK NASAL-12, 8%; most relevant: EQ-5D, 80%), while skull base meningioma patients rated 32–67% of the issues as relevant (least relevant: WHOQOL, 32%; most relevant: Sintenon’s 15D, 67%). Patients interviewed before surgery rated 17–80% of the issues in the questionnaires as relevant (least relevant: ASK NASAL-12, 17%; most relevant: EQ-5D, 80%), while patients interviewed <2 years after surgery rated 25–80% of the issues as relevant (least relevant: PCMIS and ASK NASAL-12, 25%; most relevant: EQ-5D, 80%) and patients interviewed ≥2 years after surgery rated 17–53% of the issues as relevant (least relevant: IHDNS, 17%; most relevant: NHP and VAS, 53%). See Fig. 2 for the percentage of relevant issues per questionnaire, presented for patients and HCPs, and stratified for tumor location (convexity vs. skull base) and different treatment phases (before intervention, up to 2 years after intervention and at least 2 years after intervention).

#### Issue importance

The most frequently reported HRQoL issue that was considered ‘important’ was lack of energy. This issue was reported by all patient groups and HCPs, except for patients interviewed before surgery (all patients, 42%; skull base patients, 44%; convexity patients, 40%; patients interviewed up to 2 years after surgery, 38%; patients interviewed at least 2 years after surgery, 67%; HCPs, 90%). Patients interviewed before surgery only reported issues in the physical domain as important (i.e., walking, 60%; coordination, 40%). Issues in the cognitive domain and behavior and mood domain were only reported by patients with skull base meningiomas (concentration, 44%; memory, 33%; worries, 33%), patients interviewed at least 2 years after surgery (concentration, 50%; memory, 33%; crying, 33%; nervousness, 33%) and by HCPs (memory, 60%; personality changes, 30%). Activity of daily living (ADL) issues were primarily reported to be important by convexity meningioma patients (transport, daily functioning and driving, all 30%) and patients up to 2 years after surgery (transport and daily functioning, both 38%), less frequently by skull base patients (hobbies, 33%) and not by the other (sub)groups. Further details are presented in Table 2.

**Table 2:** Most important issues as assessed by patients and health care professionals (HCPs): percentages describe the proportion of subjects in each group assessing an issue as important

Patients			
Results stratified for different treatment phase			
Before surgery (*N* = 5)	After surgery < 2 years (*N* = 8)*	After surgery ≥ 2 years (*N* = 6)	Total (*N* = 19)*
Walking: 60% Coordination: 40%	Energy: 38% Walking: 38% Instability while standing: 38% Dependence: 38% Daily functioning: 38% Transport: 38%	Energy: 67% Recurrence: 50% Concentration: 50% Pain: 33% Hearing: 33% Memory: 33% Crying: 33% Nervousness: 33%	Energy: *n* = 8 (42%)
Results stratified for different tumor locations			
Skull base (*N* = 9)*	Convexity (*N* = 10)		
Energy: 44% Concentration: 44% Headache: 33% Memory: 33% Hobbies: 33% Worries: 33%	Energy: 40% Uncertainty future: 40% Dependence: 40% Walking: 30% Coordination: 30% Transport: 30% Daily functioning: 30% Driving: 30%		
Health care professionals			
HCPs (*n* = 10)			
Energy: 90% Quality of life: 50% Memory: 60% Epilepsy: 30% Visual acuity: 30% Personality changes: 30%			

Issues are reported for each subgroup if at least 30% of subjects assessed the issue as important

^*^One skull base meningioma patient, interviewed short term after surgery, did not assess the ten most important issues

#### New relevant issues

During the semi-structured interviews with meningioma patients and HCPs, three new issues were generated by patients (loss of sensation around surgery scar, 15%; difficulty handling stress, 10%; non-visibility of the disease and its symptoms, 10%), one issue by HCPs (symptoms related to pituitary dysfunction, 30%) and one new issue by both patients and HCPs (symptoms related to executive functioning, such as multitasking: patients, 25%; HCPs, 20%).

## Discussion

The increase in use of PROMs in the last decade to measure HRQoL in meningioma patients reflects the importance of HRQoL assessment in this patient group. However, both generic and disease-specific HRQoL questionnaires used in meningioma research cover a significant array of issues that are not relevant for meningioma patients and frequently overlook relevant issues for this patient group. Moreover, patients and HCPs considered different HRQoL issues as relevant and most important. These findings support the need for a meningioma-specific PROM, measuring the construct HRQoL.

### Health-related quality of life of meningioma patients

While there is an increase in the use of PROMs to measure HRQoL in meningioma patients, the number of studies describing HRQoL data of meningioma patients is limited [44]. It is known that meningioma patients’ HRQoL before intervention is worse than that of healthy controls and depending on the HRQoL domain better or similar compared with glioma patients (all grades) [44]. HRQoL is only measured longitudinally in two studies. While about 50% of meningioma patients have an improved HRQoL after surgery in both the short (6 weeks) and long term (10–58 months), about 20% of patients have a worse HRQoL [17]. Meningioma patients receiving radiotherapy have an improved HRQoL 6 months after radiotherapy, but after 2 years of follow-up their HRQoL decreases to pre-radiotherapy levels [15]. These studies show that measuring HRQoL, in addition to conventional outcomes such as complications, resection grade, neurological complications and progression-free survival, helps to assess the effectiveness of different treatment strategies [11]. However, it is important to measure HRQoL using a questionnaire covering all aspects of HRQoL relevant to the target group. This study shows that current questionnaires, as they are not developed for meningioma patients, just partially cover relevant items for meningioma patients This could be due to the fact that many general HRQoL PROMs (e.g., SF-36, EQ-5D) do not cover disease-specific issues, and many cancer-related PROMs (e.g., EORTC QLQ-C30, FACT-G) cover issues related to side effects of systemic therapy and radiotherapy while most meningioma patients are primarily treated by surgery. Our findings therefore suggest that multiple existing questionnaires would be needed to comprehensively measure HRQoL in this patient group.

### Disagreement between patients and health care professionals

Patients and HCPs considered different HRQoL problems/issues relevant. While HCPs assessed almost all issues of all questionnaires as relevant, meningioma patients assessed many issues as non-relevant. This can be explained by the fact that HCPs have a broader knowledge of potential issues in meningioma patients, while patients only have their own situation as a reference. Another possible explanation could be that mainly HCPs, and not a sufficient number of patients, were involved in the development of some of these questionnaires. Nowadays, patients are more frequently involved in the development of new questionnaires [43]. A previous study has shown that agreement between physician- and patient-reported issues is indeed poor and that HRQoL should be patient-reported [14]. Furthermore, a disease-specific PROM, measuring the construct HRQoL, could facilitate patient-doctor communication and consequently align patient and doctor on patients’ issues and problems in clinical practice [21].

### Heterogeneity in relevance and importance of issues

Meningioma is a heterogeneous disease, as these tumors can occur at a variety of intracranial locations, possibly leading to different problems and issues. In addition, timing of assessment in studies assessing HRQoL may be important, as it is known that patients find different issues important at different treatment phases [17]. To get a comprehensive image of issues relevant and important for meningioma patients, a heterogeneous group of patients was included in this study. Indeed, we found differences in relevance and importance of issues in different subgroups based on tumor location and treatment phase. Compared with skull base meningioma patients, convexity meningioma patients rated more issues of the generic HRQoL questionnaires as relevant. Issues in the cognitive domain (e.g., concentration problems) were rated as important by skull base meningioma patients, but not by convexity meningioma patients. This is in line with previous studies that showed that skull base meningioma patients had significantly more problems than patients with a convexity meningioma in the cognitive domain (memory, verbal memory, information processing and psychomotor speed) [9, 19]. This could possibly be explained by the anatomical proximity of these tumors to the temporal lobe, which is known to support memory function [35]. In contrast, convexity meningioma patients assessed issues in the ADL domain (e.g., bathing, and driving) as important, while skull base meningioma patients did not. This may be due to the fact that convexity meningioma patients had more motor deficits (70%) than patients with a skull base meningioma (40%).

Relevance of individual HRQoL questionnaires was higher for patients interviewed before surgery than for postoperative patients, especially for those patients at least 2 years after tumor resection. Patients interviewed before surgery only reported issues in the physical domain (e.g., walking and coordination) as most important, while up to 2 years after surgery particularly problems in the ADL domain (e.g., dependence and daily functioning) were reported. Remarkably, these problems were not reported by patients interviewed after a minimum of 2 years of post-surgical follow-up, suggesting a different coping style of patients in the long term and/or psychological adjustments to chronic issues and problems [7]. Particularly issues in the cognitive domain (e.g., concentration and memory) and mood and behavior (e.g., crying and nervousness) domain were reported as “most important” by patients after a minimum of 2 years of follow-up. The long-term consequences of surgery for cognitive functioning and issues in the behavior and mood domain are unknown [23].

### Most important issue: lack of energy

The most important issue assessed by HCPs and almost all patients, except patients interviewed before surgery, was to our surprise “lack of energy” (fatigue). To our knowledge, literature on fatigue as a tumor-related symptom in meningioma patients is lacking. It is known from trials in newly diagnosed glioma patients that fatigue is a tumor-related symptom [37]. Possible underlying mechanisms of brain tumor-related fatigue in patients with primary brain tumors include activation of inflammatory pathways and disturbances of the hypothalamic and corticotropic axis [2]. Moreover, in skull base meningioma patients (all grades) receiving radiotherapy, fatigue was the most frequently reported acute and chronic symptom [18]. Studies in glioma patients have reported that 13% to 79% of patients suffer from a somnolence syndrome after radiotherapy with a peak in severity after 6 weeks [29]. In our study, only a few patients were included who received radiotherapy, so the effect of radiotherapy could not be reliably assessed. However, patients interviewed after surgery, (both <2 years and ≥2 years after surgery) rated the issue “lack of energy” as the most important issue, suggesting a possible surgical or anesthesia effect on patients’ energy levels on both the short and long term. More studies are needed to discriminate the effect of tumor type and surgery, specifically craniotomy, but also radiotherapy on patient-reported fatigue.

### Study limitations

A limitation of this study is the limited number of patients included, which hampers comparison between the different subgroups (e.g., specific tumor locations and use of anti-epileptic drugs). Patients with WHO grade II and III meningioma, as well as with neurofibromatosis type 2, were excluded. Due to the low patient number, none of the included patients had recurrent or multiple meningioma. Both issues may hamper the generalizability of the results of this study. Moreover, the issues as identified during the semi-structured interviews may be subject to the interpretation of the researcher. In addition, only issues covered by HRQoL questionnaires used in published studies including meningioma patients were included and discussed during the semi-structured interviews. Questionnaires not already used in meningioma research but of possible relevance for this patient group are missed, for instance, the MDASI and its specific brain module (MDASI-BT) and the FACT-MNG [3, 6, 45]. However, patients were also asked to report missing relevant issues during the interviews, so issues missing in existing HRQoL questionnaires were likely to be identified. Lastly, information on educational background and socio-economic status were not collected in this study, while both may influence patients’ perception on HRQoL issues [4, 32].

## Conclusions

In conclusion, existing HRQoL questionnaires are only partially relevant for meningioma patients, and they lack several relevant issues for this patient group. Agreements between patients and HCPs on issue relevance and importance were poor. Differences in the relevance of HRQoL questionnaires and importance of issues were found between convexity and skull base meningioma patients and patients interviewed before and after surgery. Therefore, the use of just one of the existing HRQoL questionnaires in studies including a heterogeneous group of meningiomas may be troublesome. Hence, we are currently developing a meningioma-specific PROM, measuring HRQoL, by including meningioma patients irrespective of cranial tumor location and treatment phase. Based on the collected data, there are multiple options for the construct and structure of the PROM. On the one hand, such a PROM may exist of just one core questionnaire, covering the majority of relevant issues for all meningioma patients, by heterogeneous patient sampling. A drawback is that this may result in a long questionnaire, increased patient burden and subsequently lower response rates. A PROM consisting of a core questionnaire covering the issues relevant to all patients, complemented by modules for certain meningioma subgroups, may resolve this problem. Which option is best will be based on the data collected in our currently ongoing study, while keeping in mind that the PROM should be relevant for the majority of meningioma patients and have a low response burden.

## Electronic supplementary material

ESM 1 (DOCX 26 kb)

## Abbreviations

ASK Nasal-12: Anterior Skull Base Nasal Inventory-12
EORTC QLQ-BN20: European Organization for Research and Treatment of Cancer Quality of Life Brain Cancer Module
EORTC QLQ-C30: European Organization for Research and Treatment of Cancer Quality of Life Core Questionnaire
EQ-5D: EuroQol 5D
FACT-BR: Functional Assessment of Cancer Therapy-Brain
FACT-G: Functional Assessment of Cancer Therapy-General
HCPs: Health care professionals
HRQoL: Health-Related Quality of Life
ICF: International Classification of Functioning, Disability and Health
IHDNS: Innsbruck Health Dimensions Questionnaire for Neurosurgical Patients
IQR: Interquartile range
MDASI: MD Anderson Symptom Inventory
MDASI-BT: MD Anderson Symptom Inventory Brain Tumor module
NHP: Nottingham Health Profile
PCMIS: Petroclival Meningioma Impairement Scale
PRISMA: Preferred Reporting Items for Systematic Reviews and Meta-Analyses
PROM: Patient-Reported Outcome Measures
Sintenon’s 15D: Sintenon’s 15-dimensional questionnaire
SF-36: 36-item Short Form Health Survey
SNAS: Sherbrooke Neuro-Oncology Assessment Scale
VAS: Visual analog scale
WHO: World Health Organization
WHOQOL: World Health Organization Quality of Life questionare
