# Supplementary material for: Health-related quality of life of cranial WHO grade I meningioma patients: are current questionnaires relevant?
Source: Acta Neurochir (Wien). 2017 Sep 27;159(11):2149–59. doi: 10.1007/s00701-017-3332-8 (PMC5636848; doi:10.1007/s00701-017-3332-8)
Supplement: Supplementary file 1 — (DOCX 26 kb) [file 701_2017_3332_MOESM1_ESM.docx]

**Health-related quality of life of cranial WHO grade I meningioma patients: are current questionnaires relevant?**

Amir H. Zamanipoor Najafabadi, Marthe C.M. Peeters, Daniel J. Lobatto, Marieke L.D. Broekman, Timothy R. Smith, Nienke R. Biermasz, Saskia M. Peerdeman, Wilo C. Peul, Martin J.B. Taphoorn, Wouter R. van Furth, Linda Dirven.

**Journal name:** Acta Neurochirurgica

**Corresponding author:** Amir H. Zamanipoor Najafabadi, *Address*: Leiden University Medical Center, Department of Neurosurgery, postal zone J11-R, Albinusdreef 2, 2333ZA Leiden, the Netherlands. *E-mail address*: a.h.zamanipoor_najafabadi@lumc.nl,
*Tel:* +31 (0) 681663780

# Supplementary Table 1 – Search strategy for MEDLINE

| **Search terms:** Meningioma, quality of life and terms to exclude studies with only animals and case reports |
| --- |
| ((("Meningioma"[MesH] OR "Meningioma"[Tw] OR "Meningiomas"[Tw] OR "Meningiomatosis"[Tw] OR "Meningiomatoses"[Tw] OR "Meningeal Neoplasms"[MeSH] OR "Meningeal Neoplasms"[Tw] OR "Meningeal Neoplasm"[Tw]) **AND** ("Quality of Life"[mesh] OR "Health Surveys"[mesh] OR "Questionnaires"[Mesh] OR "Self Report"[mesh] OR "Patient Outcome Assessment"[mesh] OR "Health Status Indicators"[mesh] OR "Quality of Life"[tw] OR "QoL"[tw] OR "HRQL"[tw] OR "HRQOL"[tw] OR "PQoL"[tw] OR "AQoL"[tw] OR "subjective wellbeing"[tw] OR "subjective well-being"[tw] OR "Patient Reported Outcome"[tw] OR "Patient Reported Outcomes"[tw] OR "patient reported"[tw] OR "PRO"[tw] OR "PROs"[tw] OR "PROM"[tw] OR "PROMs"[tw] OR "health survey"[tw] OR "health surveys"[tw] OR "Questionnaires"[tw] OR "questionnaire"[tw] OR "Self reports"[tw] OR "Self report"[tw] OR "Self-reported"[tw] OR "Patient Outcome Assessments"[tw] OR "Patient Outcome Assessment"[tw] OR "health status indicator"[tw] OR "health status indicators"[tw] OR health status indicat*[tw] OR "outcome instrument"[tw] OR "outcome instruments"[tw] OR "health score"[tw] OR "health scores"[tw] OR health scor*[tw])) **NOT** ("Animals"[mesh] NOT "Humans"[mesh]) NOT ("Case Reports"[pt] NOT "Clinical Trial"[pt])) |

**The search strategy was adapted for the following electronical databases: Embase, Web of Science, CINAHL, PsychInfo, Academic Search Premier, COCHRANE and ScienceDirect.**

**Supplementary Table 2 – Subject characteristics: different treatment phase**

|  | **All patients (n=20)** | **Before surgery (n=5)** | **After Surgery < 2 years (n=9)** | **After surgery ≥ 2 years (n=6)** | ***p*-value** |
| --- | --- | --- | --- | --- | --- |
| **Age in years at interview, median (range)** | 57 (39-73) | 67 (43-69) | 56 (44-68) | 55 (39-65) | 0.73 |
| **Sex, n (% female)** | 15 (75%) | 4 (80%) | 7 (78%) | 4 (67%) | 0.85 |
| **Tumor Location, n (%)** |  |  |  |  | 0.61 |
| **Convexity** | 10 (50%) | 3 (60%) | 5 (56%) | 2 (33%) |  |
| **Skull base** | 10 (50%) | 2 (40%) | 4 (44%) | 4 (67%) |  |
| **Karnofsky Performance Status, median (range)** | 95  (70-100) | 100  (70-100) | 90  (70-100) | 100 (80-100) | 0.11 |
| **Charlson Comorbidity Index, n (%)** |  |  |  |  | 0.15 |
| **0** | 15 (75%) | 4 (80%) | 5 (56%) | 6 (100%) |  |
| **1-2** | 4 (20%) | 1 (20%) | 3 (33%) | 0 (0%) |  |
| **>2** | 1 (5%) | 0 (0%) | 1 (11%) | 0 (0%) |  |
| **Midline shift present, n (%)** | 4 (20%) | 1 (20%) | 2 (22%) | 1 (17%) | 0.96 |
| **Edema present, n (%)** | 16 (80%) | 5 (100%) | 7 (78%) | 4 (67%) | 0.38 |
| **Corticosteroid use, n (%)** | 3 (15%) | 2 (40%) | 1 (11%) | 0 (0%) | 0.16 |
| **Antiepileptic drug use, n (%)** | 3 (15%) | 1 (20%) | 1 (11%) | 1 (17%) | 0.90 |
| **Surgical resection, n (%)** | 15 (75%) | - | 9 (100%) | 6 (100%) | 0.70 |
| **Simpson grade I** | 7 (35%) | - | 5 (56%) | 2 (33%) |  |
| **Simpson grade II** | 6 (30%) | - | 3 (33%) | 3 (50%) |  |
| **Simpson grade III** | 0 (0%) | - | 0 (0%) | 0 (0%) |  |
| **Simpson grade IV** | 2 (10%) | - | 1 (11%) | 1 (17%) |  |
| **Simpson grade V** | 0 (0%) | - | 0 (0%) | 0 (0%) |  |
| **Patients with Surgical complications, number (%)** | 4 (20%) | 1 (17%) | 1 (11%) | 2 (40%) | 0.71 |
| **Number of infections** | 3 (15%) | 0 (0%) | 1 (11%) | 2 (40%) |  |
| **Number of cardiovascular complications** | 1 (5%) | 1 (17%) | 1 (11%) | 0 (0%) |  |
| **Number of neurological complications** | 1 (5%) | 0 (0%) | 0 (0%) | 0 (0%) |  |
| **Number of pulmonal complications** | 1 (5%) | 0 (0%) | 0 (0%) | 1 (20%) |  |
| **Postsurgical radiotherapy, n (%)** | 2 (10%) | 0 (0%) | 1 (11%) | 1 (17%) | 0.65 |

**n: number.**

Supplementary Table 3 - Subject characteristics: different tumor location

|  | **All patients (n=20)** | **Convexity (n=10)** | **Skull base (n=10)** | ***p*-value** |
| --- | --- | --- | --- | --- |
| **Age in years at interview, median (IQR)** | 57 (48-67) | 62 (53-68) | 50 (44-66) | .06 |
| **Sex, number (% female)** | 15 (75%) | 7 (70%) | 8 (80%) | 1.00 |
| **Time since clinical diagnosis in months, median (IQR)** | 23 (5-51) | 18 (4-55) | 30 (5-59) | .74 |
| **Karnofsky Performance Status, median (IQR)** | 95 (80-100) | 95 (90-100) | 95 (80-100) | .74 |
| **Charlson Comorbidity Index, n (%)** |  |  |  | .59 |
| **0** | 15 (75%) | 8 (80%) | 7 (70%) |  |
| **1-2** | 4 (20%) | 2 (20%) | 2 (20%) |  |
| **>2** | 1 (5%) | 0 (0%) | 1 (10%) |  |
| **Midline shift present, n (%)** | 4 (20%) | 2 (20%) | 2 (20%) | 1.00 |
| **Edema present, n (%)** | 16 (80%) | 7 (70%) | 9 (90%) | .58 |
| **Corticosteroid use, n (%)** | 3 (15%) | 2 (20%) | 1 (10%) | 1.00 |
| **Antiepileptic drug use, n (%)** | 3 (15%) | 2 (20%) | 1 (10%) | 1.00 |
| **Moment of interview** |  |  |  | .61 |
| **Before surgery** | 5 (25%) | 3 (30%) | 2 (20%) |  |
| **After surgery < 2 year** | 9 (45%) | 5 (50%) | 4 (40%) |  |
| **After surgery ≥ 2 years** | 6 (30%) | 2 (20%) | 4 (40%) |  |
| **Surgical resection, n (%)** | 15 (75%) | 7 (70%) | 8 (80%) | .69 |
| **Simpson grade I** | 7 (35%) | 4 (40%) | 3 (30%) |  |
| **Simpson grade II** | 6 (30%) | 2 (20%) | 4 (40%) |  |
| **Simpson grade III** | 0 (0%) | 0 (0%) | 0 (0%) |  |
| **Simpson grade IV** | 2 (10%) | 1 (10%) | 1 (10%) |  |
| **Simpson grade V** | 0 (0%) | 0 (0%) | 0 (0%) |  |
| **Patients with Surgical complications, n (%)** | 4 (20%) | 2 (20%) | 2 (20%) | 1.00 |
| **Number of infections** | 3 (15%) | 1 (10%) | 2 (20%) |  |
| **Number of cardiovascular complications** | 1 (5%) | 1 (10%) | 0 (0%) |  |
| **Number of neurological complications** | 1 (5%) | 0 (0%) | 1 (10%) |  |
| **Number of pulmonal complications** | 1 (5%) | 0 (0%) | 1 (10%) |  |
| **Postsurgical radiotherapy, n (%)** | 2 (10%) | 2 (20%) | 0 (0%) | 0.24 |

n: number. IQR: interquartile range
